# Enhanced distance-dependent fluorescence quenching using size tuneable core shell silica nanoparticles†

**DOI:** 10.1039/c8ra05929b

**Published:** 2018-10-19

**Authors:** Mohamed M. Elsutohy, Amjad Selo, Veeren M. Chauhan, Saul J. B. Tendler, Jonathan W. Aylott

**Affiliations:** Boots Science Building, School of Pharmacy, University Park Campus, University of Nottingham Nottingham NG7 2RD UK jon.aylott@nottingham.ac.uk; Department of Pharmaceutical Analytical Chemistry, Faculty of Pharmacy, Al-Azhar University Assiut 71524 Egypt; Vice-Chancellor's Department, University of York Heslington York YO10 5DD UK

## Abstract

Silica nanoparticles (SNPs) have been used as favoured platforms for sensor, drug delivery and biological imaging applications, due to their ease of synthesis, size-control and bespoke physico-chemical properties. In this study, we have developed a protocol for the synthesis of size-tuneable SNPs, with diameters ranging from 20 nm to 500 nm, through the optimisation of experimental components required for nanoparticle synthesis. This protocol was also used to prepare fluorescent SNPs, *via* covalent linkages of fluorophores, to the nanoparticle matrix using 3-aminopropyltriethoxysilane (APTES). This enabled the fabrication of ratiometric, fluorescent, pH-sensitive nanosensors (75 nm diameter) composed SNPs covalently linked to two pH-sensitive fluorescent dyes Oregon Green (OG) and 5(6)-carboxyfluorescein (FAM) and a reference fluorescent dye 5-(6)-carboxytetramethylrhodamine (TAMRA), extending the dynamic range of measurement from pH 3.5 to 7.5. In addition, size-tuneable, core–shell SNPs, covalently linked to a fluorescent TAMRA core were synthesised to investigate distance-dependant fluorescence quenching between TAMRA and black hole quencher 2 (BHQ2®) using nanometre-sized silica shells as physical spacers. The results showed a significant fluorescence quenching could be observed over greater distances than that reported for the classical distance-dependent molecular fluorescence quenching techniques, *e.g.* the Förster (fluorescence) resonance energy transfer (FRET). The methods and protocols we have detailed in this manuscript will provide the basis for the reproducible production of size tunable SNPs, which will find broad utility in the development of sensors for biological applications.

## Abstract 2

Core–shell silica nanoparticles were shown to demonstrate quenching between a fluorescent core and surface black hole quencher layer, separated by a size controllable silica shell.

## Graphical abstract

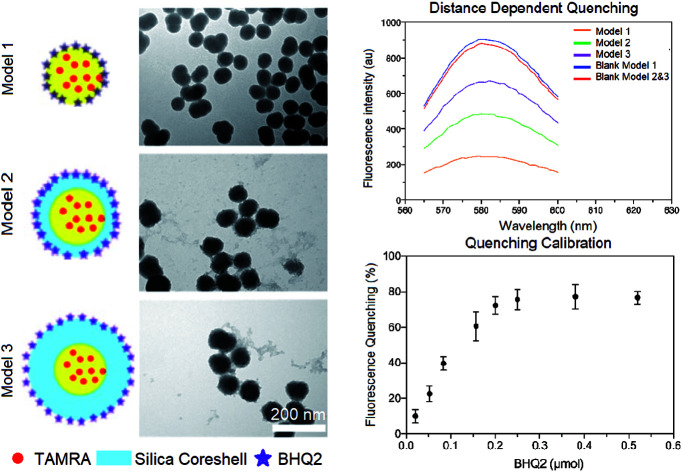

## Introduction

Nanoparticle-based techniques have opened new horizons towards the development of smart tools for many applications, including drug delivery, biological imaging and intracellular measurements.^1^

**Fig. 1:**
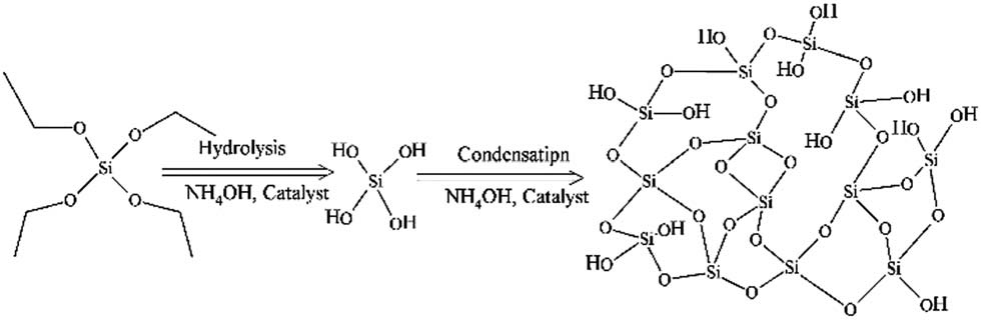
A diagram illustrates the Stöber method for the synthesis of SNPs *via* hydrolysis and condensation of silica precursor (TEOS) using ammonium hydroxide (NH_4_OH) as a catalyst.

In particular, silica nanoparticles (SNPs) have been reported in a variety of studies due to their ease of synthesis, biocompatibility and versatile physicochemical properties.^2,3^ Synthesis of SNPs was firstly described by Stöber and co-workers^4^ in 1968 *via* hydrolysis and condensation of a silane alkoxide precursor, usually alcoholic tetraethylorthosilicate (TEOS), using ammonium hydroxide (NH_4_OH) as a catalyst, Fig. 1.

During nanoparticle synthesis, a variety of components such as fluorescent molecules, drugs or sensing elements can be incorporated into the nanoparticle matrix to produce functionalised SNPs.^5–8^ For example, fluorescent SNPs that have been reported for biological imaging and sensing could be synthesised *via* covalent binding of fluorescent dye molecules to the nanoparticle matrix using a chemically active silane compound, 3-aminopropyltriethoxysilane (APTES) as a linker.^9^ In addition, the nanoparticle surface can be readily functionalised with several chemical groups to facilitate further surface conjugation, sensing and targeted therapy.^10,11^

Whilst many protocols have been reported for the synthesis of SNPs, there is a marked variation in the reported nanoparticle size from one study to another, based on the type and amount of reactant or catalyst used.^12–15^ According to Stöber, different-sized SNPs can be fabricated through optimisation of the quantity of each reactant and catalyst required for nanoparticle synthesis. However, it is important to note previously reported studies^12,16^ do not agree with the findings reported by Stöber. These contradicting findings indicate the need for further studies to develop and optimise a valid protocol that can be readily followed for the synthesis of size-tuneable SNPs, especially for applications where nanoparticle size control is critical, *e.g.* intracellular measurements.

**Fig. 2:**
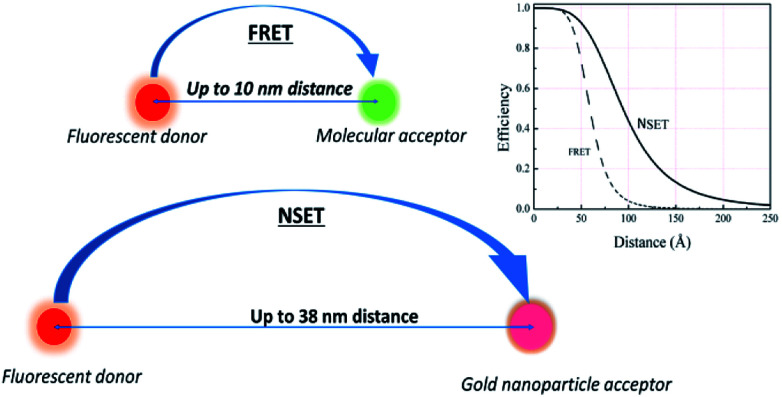
Diagrammatic representation of the maximum distance reported for efficient Förster resonance energy transfer (FRET), occurring between molecular donor–acceptor system, and nanometal surface energy transfer (NSET), observed between a fluorescent donor and gold nanoparticle.

Furthermore, due to the ease of chemical functionalisation, silica has been used as an inert material to coat many systems, *e.g.* quantum dots and gold nanoparticles, producing core–shell structures surrounded by silica layers that can be readily functionalised.^17,18^ These structures have been used to study the interaction between a fluorescent donor and an acceptor/quencher, separated by nanometre-controlled distances, using the phenomena of the Förster (fluorescence) resonance energy transfer (FRET) and nanometal surface energy transfer (NSET),^5,19^Fig. 2.

Changes in the recorded fluorescence signals of donor–acceptor systems have been utilised as a sensing strategy in biosensor technology, viral diagnostics and for determination of distances between macromolecules (spectroscopic rulers).^20–24^ According to the literature, for an efficient FRET or NSET to occur the maximum distance between the fluorescent donor and its corresponding acceptor must be less than 10 or 20 nm, respectively.^25–27^ However, recent studies have shown that an efficient fluorescence transfer/quenching was observed over longer distances than that reported.^28–30^ For example, Reineck *et al.* reported that NSET was observed between fluorophore molecules and gold nanoparticles over a distance of 38 nm using silica as physical spacers.^31^ This fluorescence transfer occurred when a silica shell was used as a separator between the donor and its acceptor; nevertheless, this fluorescence energy transfer/quenching was attributed to the plasmonic properties of gold nanoparticles due to the localised surface plasmon resonance (LSPR), excluding any potential role that silica could exhibit. This reflects the fact that further research is required to investigate fluorescence transfer/quenching between a fluorescent donor and a quencher over distances using silica shells as spacers.

In this study, we have developed a protocol optimising the amounts of each reactant and catalyst required for nanoparticle synthesis to produce size-controlled and tuneable SNPs. This protocol was applied to synthesise fluorescent SNPs covalently bounded to fluorophores using APTES. Ratiometric, fluorescent, pH-sensitive nanosensors and core–shell fluorescent SNPs were also fabricated. Fluorescence quenching between 5-(6)-carboxytetramethylrhodamine (TAMRA) and black hole quencher 2 (BHQ2®), separated by silica shells with different thickness was observed and quantified.

## Results and discussion

### Synthesis of size-controlled/tuneable SNPs

Previously studies protocols have attempted to prepare SNPs; however, it was challenging to reproduce the reported particle diameters.^13,14,32^ Therefore, the primary aim in our study was to develop a protocol to optimise the synthesis of size-tuneable SNPs that can be used in applications where nanoparticle size-control is important. The amounts of reactants and catalysts produces SNPs with variable sizes. ^33^ Therefore, we started our study by investigating the influence of controlling the quantities of each reactant and catalyst (TEOS, NH_4_OH and ethanol) on the resulting nanoparticle diameter.

The interplay between varying volumes of TEOS (180–2000 μL, 98%, equivalent to 0.80 to 8.8 mmol L^−1^) and nanoparticle diameter was investigated without changing the volume of NH_4_OH (1.0 mL of 28–30% v/v, 7.7 mmol L^−1^) and ethanol (absolute 99%, 16.75 mL). The hydrodynamic diameter of each nanoparticle batch (*n* = 5) was measured using dynamic light scattering (DLS).

**Fig. 3:**
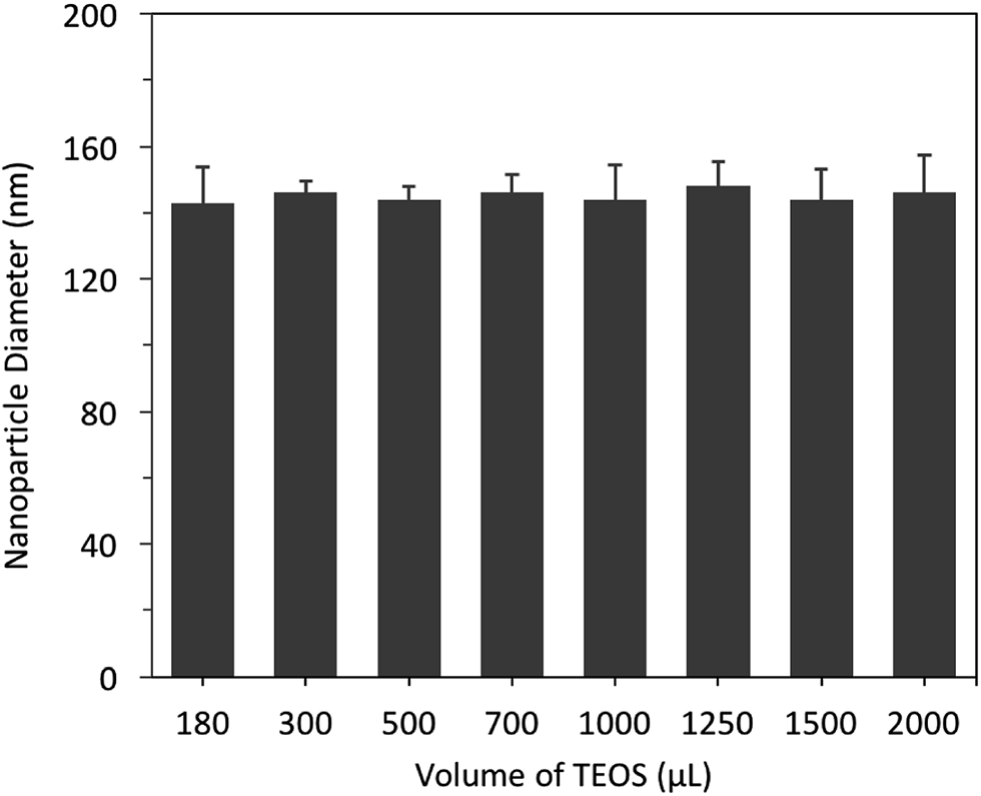
The effect of controlling the volumes of TEOS (180–2000 μL, 98%, 0.80 to 8.8 mmol L^−1^) on the hydrodynamic diameter of SNPs, measured using DLS (*n* = 5).

The results showed that different volumes of TEOS did not significantly (*p* > 0.05) influence the mean nanoparticle diameter, which were centred at 145 ± 14 nm (polydispersity index, (PDI) ≤ 1.04), Fig. 3. In addition, the recovered nanoparticles (100 ± 20 mg) were not influenced by increasing the volume of TEOS. As a result, 500 μL (2.2 mmol L^−1^) of TEOS was selected for all further experiments. In comparison, previously reported studies showed that increasing the concentration of TEOS resulted in an increase or occasionally a decrease in the nanoparticle diameter.^12,16^ The results obtained in this study are in agreement with that reported by Stöber, who concluded that varying amounts of TEOS did not influence the nanoparticle size.^4^

**Fig. 4:**
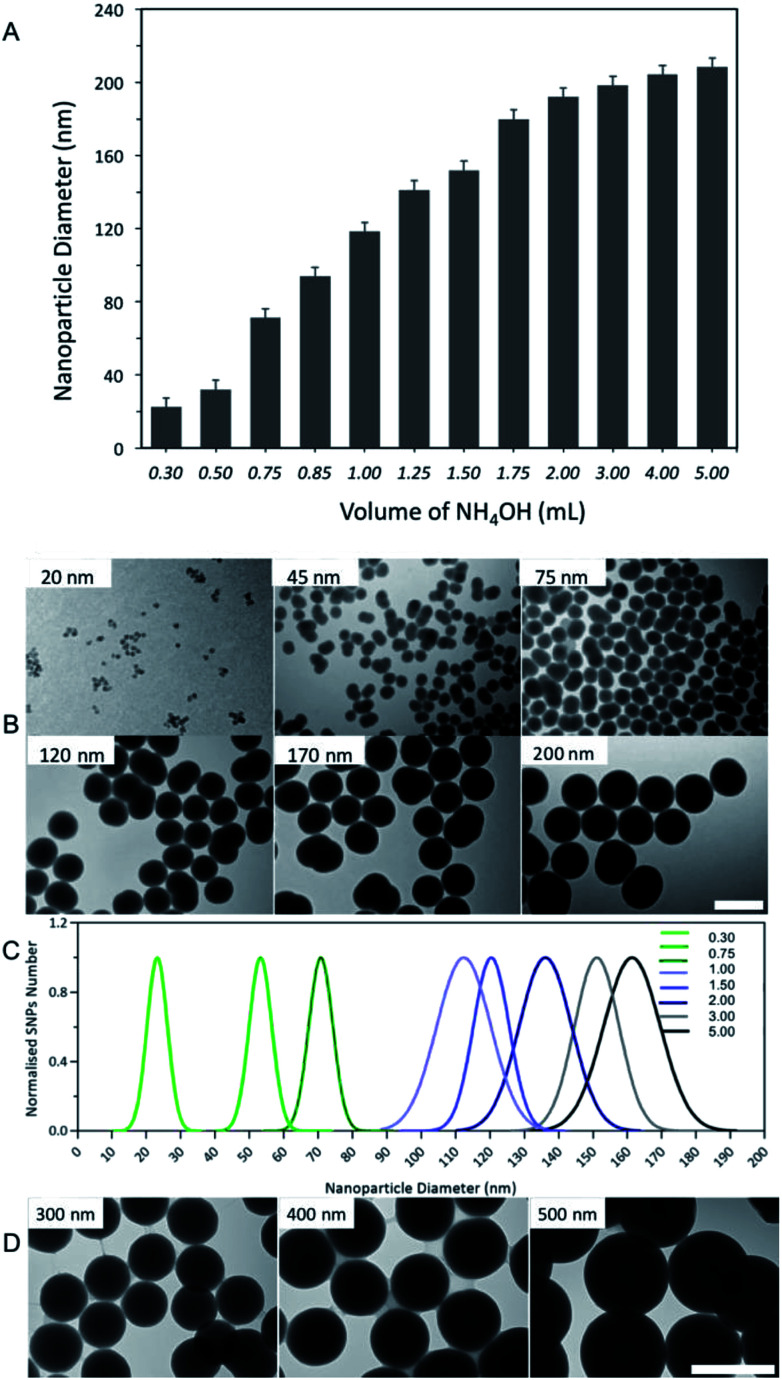
(A) The interplay between different volumes of NH_4_OH (0.3 to 5.0 mL (28–30%), 2.3 to 38.5 mmol L^−1^) and SNPs diameter, measured by a DLS (*n* = 5). (B) TEM images of selected SNPs samples, produced using different volumes of NH_4_OH, scale bar = 200 nm. (C) TEM size distribution histogram of the resulting SNPs batches (D) TEM images of SNPs with diameters ranging from 200 to 500 nm, synthesised using the optimised protocol combined with the seed growth method,^35^ scale bars = 500 nm.

In contrast, increasing the volumes of NH_4_OH (28–30% v/v) from 0.3 to 5.0 mL (2.3 to 38.5 mmol L^−1^), whilst keeping the quantities of all other reactants constant (optimised TEOS of 500 μL, 2.2 mmol L^−1^, and 16.75 mL of ethanol), gradually increased nanoparticle diameters from 20 nm to 200 nm, Fig. 4A. Furthermore, by increasing the volume of NH_4_OH beyond 2.0 mL (15.4 mmol L^−1^) did not significantly (*p* > 0.05) influence the nanoparticle diameter as it remained at ∼200 nm.

With respect to nanoparticle yield, increasing the NH_4_OH volume increased the mass of nanoparticles recovered (see Table 1 in the ESI†). Therefore, for the efficient separation of SNPs with a diameter < 50 nm membrane dialysis was used, whereas, freeze-drying with centrifugation was used to collect SNPs > 50 nm.

DLS is an excellent methodology to determine nanoparticle sizes, nevertheless, it is important to combine more than one technique to further information on nanoparticle characteristics and confirm the results obtained from the DLS measurements.^34^ Therefore, transmission microscopy (TEM) was used to measure the nanoparticles size distribution and determine their morphology. Fig. 4B and C, shows the results obtained from TEM imaging and indicate that SNPs produced using different volumes of NH_4_OH were spherical in shape and had a narrow size distribution for all batches. The size measured using TEM was slightly smaller than that obtained from the DLS measurements. This is an expected observation as DLS measures the hydrodynamic diameter, which is the nanoparticle size combined with a surrounding solvent layer.^36^ Taken together, the results obtained from DLS agreed with TEM imaging.

To synthesise SNPs with a diameter greater than 200 nm we optimised a synthesis method that controls the volumes of NH_4_OH (0.3–5.0 mL of 28–30% v/v) with constant volumes of TEOS (μL) and ethanol (16.75 mL), in addition to the seed growth method that reported by Bogush *et al.*^35^ This procedure was based on a second addition of TEOS (1.5 mL, 6.6 mmol L^−1^) to the reaction mixture after the first 24 hours of the nanoparticle synthesis, prepared by using the different volumes of NH_4_OH. The results showed that this two-step synthesis protocol produced monodisperse SNPs with diameters ranging from 200 to 500 nm (PDI < 0.10), Fig. 4D.

**Fig. 5:**
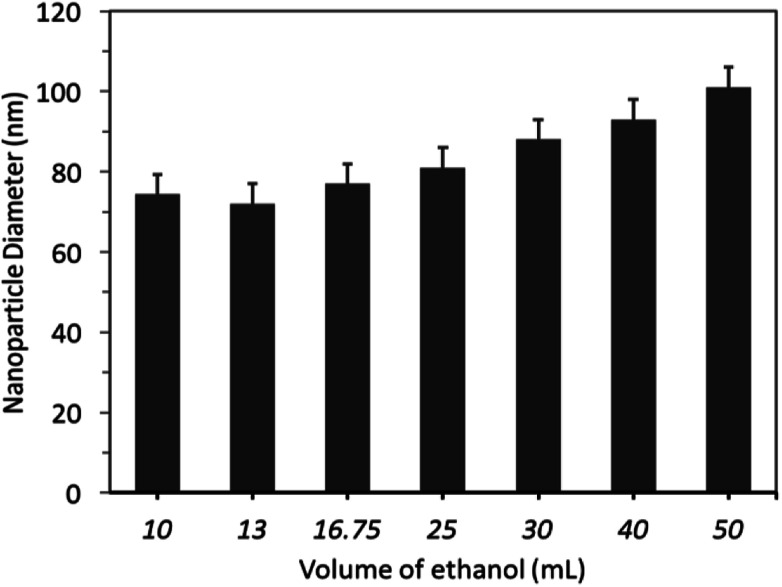
The effect of different volumes of ethanol (10–50 mL) on SNPs diameter.

The effect of different volumes of alcohol solvent (ethanol) on nanoparticle diameter and size distribution were investigated. Firstly, SNPs were prepared by using variable volumes of ethanol, ranging from 10 mL to 50 mL, and constant volumes of TEOS (500 μL) and NH_4_OH (0.75 mL). The results showed that increasing the volume of ethanol from 10 to 25 mL did not significantly (*p* > 0.05) influence the nanoparticle diameter, which remained at 70 ± 18 nm (PDI ≤ 0.052), Fig. 5. By increasing the volume of ethanol beyond 25 mL, but less than 50 mL, subtly increased the nanoparticle diameter.

Secondly, the effect of alcohol type on nanoparticle diameter and size distribution was studied using either methanol or isopropanol (16.75 mL) instead of ethanol. The results revealed that using methanol for nanoparticle synthesis reduced nanoparticle size to 65 nm. This diameter was smaller than that obtained using the same amount of ethanol (75 nm), however, methanol was excluded from further experiments due to potential toxicity and harmful environmental effects.^37^ Furthermore, polydisperse, large SNPs with a diameter of 220 nm and wide-size distribution were produced when isopropanol was used for nanoparticle synthesis. This could reflect the potential effect of alcohol polarity on nanoparticle size, aggregation and shape.^38,39^ Therefore, we used ethanol (16.75 mL) as an alcoholic medium for nanoparticle synthesis.

Synthesis of SNPs can be explained by the von Weimarn and Ostwald ripening theories that describe how particles with a specific size are formed.^40,41^ According to these theories, for any particle to be formed, a supersaturated solution of the corresponding ions/molecules, hydrolysed TEOS in this case, is required to produce primary unstable small nuclei (nucleation). Once these primary nuclei reach equilibrium, they combine to form secondary, thermodynamically stable larger nuclei which spontaneously grow to form more stable and uniform particles. During SNPs synthesis, NH_4_OH is used as a catalyst to initiate the hydrolysis and condensation of TEOS, thus, using a constant volume of NH_4_OH with varying quantitates of TEOS does not influence the nanoparticle diameter as the number of the hydrolysed TEOS molecules is limited by the amount of NH_4_OH. In contrast, increasing the quantity of NH_4_OH increases the rates of hydrolysis (nucleation) and condensation (particle growth), which in turn increases the number of hydrolysed TEOS molecules required for the primary nuclei formation and thus, increasing nanoparticle diameter, which could improve particle yield.

### Production of fluorescent SNPs and pH-sensitive nanosensors

**Fig. 6:**
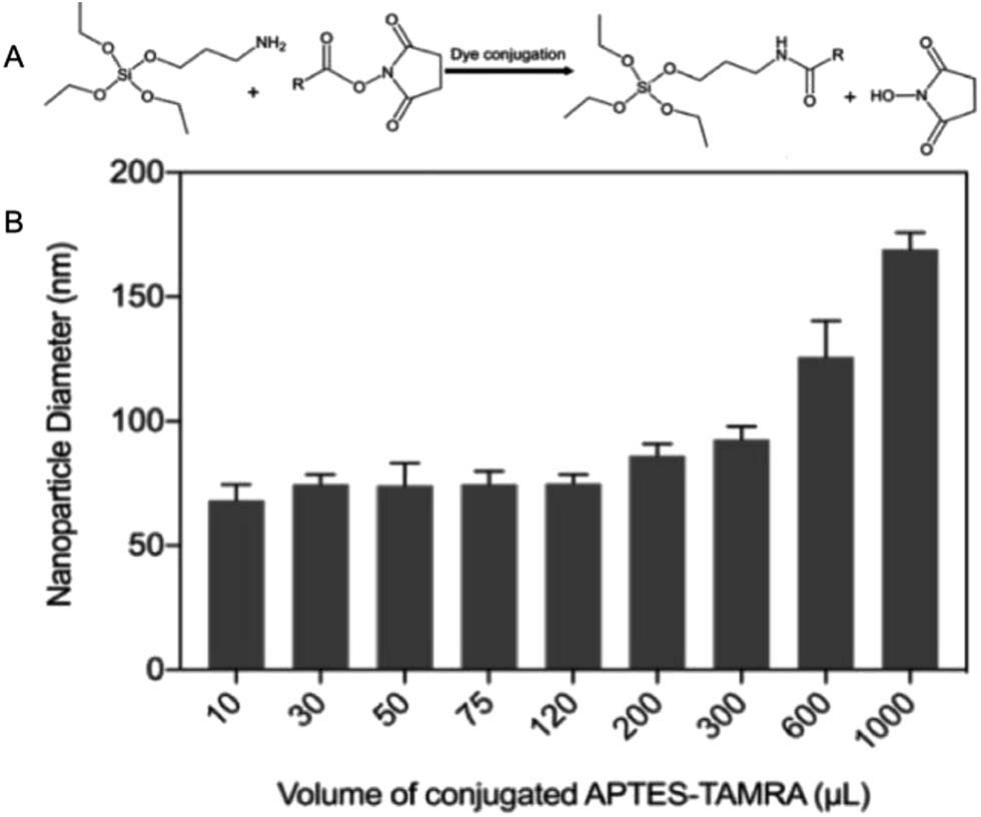
(A) A schematic diagram shows the covalent conjugation reaction between the succinimidyl ester derivative of a fluorophore (*R*) to APTES to prepare a conjugate that used for the synthesis of fluorescent SNPs. (B) The effect of APTES–TAMRA conjugate on nanoparticle diameter.

Fluorescent SNPs (75 ± 8 nm) were produced *via* covalent binding of fluorescent dye molecules to the nanoparticle matrix. Prior to nanoparticle synthesis, the succinimidyl ester derivative of fluorescent dye molecules were covalently linked to APTES (Fig. 6A) in an excess molar ratio of APTES : fluorescent dye (1.2 : 1) to ensure that all fluorophores were successfully conjugated to APTES.^42,43^ APTES-fluorescent dye conjugates were added during nanoparticle synthesis to facilitate covalent linking of the fluorescent dye molecules to SNPs matrix and produce fluorescent SNPs. This experimental study was initiated by using 5-(and-6)-carboxytetramethylrhodamine (TAMRA) as an exemplar fluorophore to investigate the effect of varying volumes of APTES–fluorophore conjugate on nanoparticle diameter. The results showed that the hydrodynamic diameters of fluorescent SNPs were 75 ± 13 nm for all batches, similar to that obtained with the non-fluorescent SNPs, provided that the volume of this conjugate were less than 300 μL. Further increase in TAMRA–APTES above volume greater than 300 μL, up to 1000 μL, increased the nanoparticle diameter to ∼180 nm, Fig. 6B.

In addition, the procedure, described for the synthesis of fluorescent SNPs, was also utilised to fabricate ratiometric, fluorescent, pH-sensitive, silica-based nanosensors. These nanosensors were composed SNPs (75 nm diameter) covalently linked to two pH-sensitive fluorescent dyes, Oregon Green (OG) and 5-(6)-carboxyfluorescein (FAM) in combination with TAMRA as a reference pH-insensitive fluorophore to permit ratiometric measurements. Combination of such two pH-sensitive fluorophores and optimising their ratio extended the effective dynamic range of nanosensors from pH 3.5 to 7.5 ^44^ (see ESI† for further details).

### Fluorescence-quenching study using size-controlled fluorescent core–shell SNPs

**Fig. 7:**
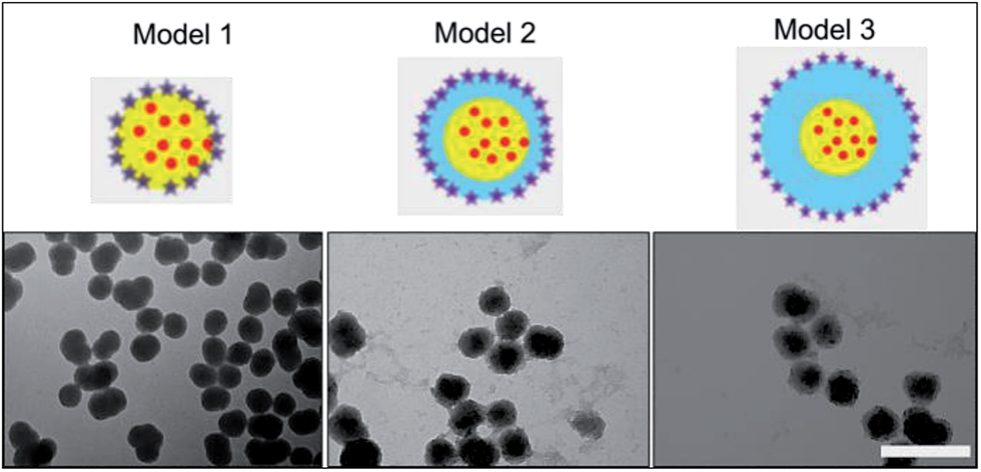
Diagrammatic representation of model 1, model 2 and 3 and their corresponding TEM images. Red dots within the yellow core illustrates the 75 TAMRA-SNPs while the stars on the surface express BHQ2 molecules, linked to nanoparticle surface, the blue layer illustrates the plain non-fluorescent silica shell surrounding the fluorescent core with thickness of ∼12.5 nm and 25 nm for model 2 & 3 respectively. Scale bar = 200 nm.

Silica has been previously reported as a spacer (shell) between a fluorescent molecule (donor) and noble-metal nanoparticles, such as gold, (acceptor or quencher) to investigate NSET or distance-dependent fluorescence transfer/quenching.^26,27,31^ To the best of our knowledge, molecular fluorescence quenching/transfer between a fluorescent dye and black hole quenchers (BHQs) using silica spacers has not yet been studied. In this study, we conducted research to investigate fluorescence quenching between TAMRA (in the core) as a fluorescent emitter molecule and a corresponding molecular quencher (BHQ2) (linked to the surface) over different distances using silica shells as separators. BHQ2 was chosen as a selective molecular quencher for TAMRA, because it absorbs light at the same wavelength range of TAMRA fluorescence without re-emission of the absorbed light.^45^ Three different models of TAMRA-SNPs and core–shell TAMRA-SNPs were synthesised and characterised, Fig. 7. Model 1 was composed of 75 nm TAMRA-labelled silica core. This fluorescent core was also used to synthesise model 2 and model 3 that were composed of a TAMRA labelled core (model 1) surrounded by non-fluorescent silica with thickness of 12.5 nm and 25 nm, respectively (Fig. 7), as measured by TEM and confirmed by DLS measurements. Binding of BHQ2 to the nanoparticle model surface was performed using the EDC/NHS chemistry of molecular conjugation (see ESI†).

To study the fluorescence quenching over the specified separation distances of the developed silica models, the fluorescence signal of each model was measured in comparison to fluorescent TAMRA-SNPs that were not linked to BHQ2. The percentage of fluorescence quenching was calculated using eqn (1):1
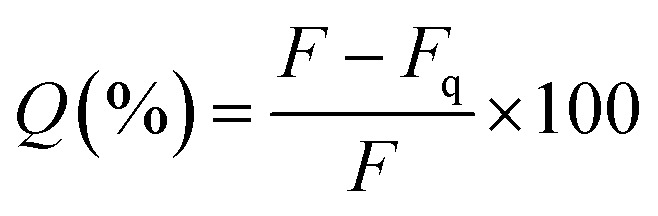
where *Q* is the percentage of fluorescence quenching, *F* is the fluorescence intensity of TAMRA-SNPs (without BHQ2), and *F*_q_ is the fluorescence intensity after modification and binding to BHQ2.

**Fig. 8:**
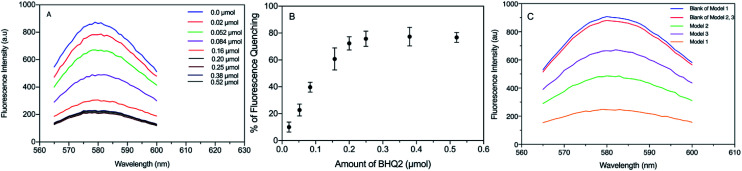
(A) Fluorescence spectra of model 1, linked to different concentrations of BHQ2 and compared to a blank (without BHQ2 molecules). (B) Calibration curve for the percentage of fluorescence quenching resulted from model 1 (*n* = 5) and a range of BHQ2 concentrations. (C) Fluorescence spectra of model 1, 2 and 3 after binding to BHQ2 (0.25 μmol L^−1^), compared to a corresponding blank (SNPs model prepared without BHQ2).

The results showed that fluorescence quenching of TAMRA was observed in all models after binding to BHQ2, compared to the corresponding blank sample of each model, Fig. 8. While the relationship between BHQ2 concentration and fluorescence quenching of TAMRA was established using model 1 using different concentrations of BHQ2, linked to nanoparticle surface. This relationship was similar to a typical Beer–Lambert calibration curve, which indicates that fluorescence quenching is dependent on BHQ2 concentration while further increase in BHQ2 quantity above 0.20 μmol L^−1^ did not influence the percentage of TAMRA quenching (Fig. 8A). As a result, 0.25 μmol L^−1^ of BHQ2 was selected as the optimised concentration that provided maximum fluorescence quenching of TAMRA (Fig. 8B). The maximum fluorescence quenching of model 1, model 2 or model 3 was ∼75%, 45% or 25% respectively, (Fig. 8C).

Further investigations were performed to study the possibility of whether BHQ2 diffusion through the silica matrix could interfere with the obtained fluorescence quenching. To conduct this experiment, model 1 was prepared without any surface functionalisation or activation, required to bind BHQ2, and stirred overnight with BHQ2 (0.25 μmol L^−1^). After nanoparticle separation, the fluorescence signal of TAMRA was detected and compared to that recorded for the corresponding nanoparticle sample, treated similarly without mixing with BHQ2. The results revealed that the fluorescence intensity of TAMRA of the non-functionalised model 1 and blank sample were not statistically different (*p* > 0.05) (Fig. S4, ESI†). This indicates minimal diffusion of BHQ2 through the silica matrix and confirms that observed fluorescence quenching of the developed models is due to BHQ2 that is chemically linked to the nanoparticle surface and such variable quenching can be due to changes in the separation distance.

In our systems, the fluorescence quenching observed in model 1 (75%, no spacer layer) fits with FRET theory where the distance between donor and acceptor is <10 nm. We would anticipate some fluorescence being emitted from the model 1 core as the average particle size is 75 nm and so not all fluorophores will be within the FRET distance limits. In addition to fluorescence quenching there may be a contribution to the reduced fluorescence output *via* re-absorption of TAMRA emission by BHQ2. Our findings for models 2 & 3 highlight that fluorescence quenching occurs beyond the 10 nm distance limit accepted for efficient fluorescence resonance energy transfer (FRET) between donor and quencher molecules^5,18,45^ (Fig. 2). For our engineered SNPs (models 2 & 3) where TAMRA and BHQ2 were separated by silica shells with thickness of approximately 12.5 and 25 nm, respectively, conventional FRET theory does not explain the quenching observed as the separation distance is greater than 10 nm. While energy transfer between a fluorescent donor and a gold nanoparticle quencher across a silica spacer layer has previously been observed over an extended distance, this has been attributed to NSET.^30^ Taken together, we postulate that the observed quenching is due to a combination of energy transfer, emission re-absorption and a possible influence of the silica shell (similar to that hypothesised by Reineck *et al.*^31^).

While the mechanism behind this observed distance-dependent fluorescence quenching has not yet been confirmed, fluorescence quenching over this extended distance could provide a new sensing strategy where varying the distance between the fluorescence donor–acceptor system is used for detection. This could include the monitoring of DNA hybridisation and the development of aptamer-based biosensors (aptasensors) for the detection of biomarkers, *e.g.* ATP, thrombin and adenosine.^46^

## Conclusion

In this study, a simple protocol for the synthesis of size-tuneable SNPs, with diameters ranging from 20 to 500 nm, has been developed by optimising the amount of each reactant and catalyst that are required for nanoparticle synthesis. The results showed that NH_4_OH plays a key role in controlling the size of SNPs and by careful selection of the volume of NH_4_OH the nanoparticle diameter can be varied from 20 nm to 200 nm. Through selection of optimised NH_4_OH volumes and subsequent addition of TEOS, the nanoparticle diameters were controllably increased from 200 nm to 500 nm. In addition, SNPs covalently linked to fluorescent dye molecules were produced. This enabled the fabrication of ratiometric, fluorescent pH-sensitive nanosensors. Furthermore, using the versatile chemistry and size-tunability of SNPs, three models of fluorescent-quencher SNPs were synthesised and used to investigate fluorescence quenching between fluorophore molecules (TAMRA) and a molecular black hole quencher (BHQ2). The results showed a significant distance-dependent fluorescence quenching over a greater distance than that expected, which could be used as an analytical technique in sensing applications, *e.g.* nanosensors where the fluorescence signal is distant dependent.

## Experimental

### Materials and chemicals

The succinimidyl ester derivatives of Oregon Green® 488 carboxylic acid 5-isomer (OG), 5-(6)-carboxyfluorescein (FAM) and 5-(and-6)-carboxytetramethylrhodamine (TAMRA) were obtained from Invitrogen, USA. Tetraethylorthosilicate (TEOS, 99%), 3-aminopropyltriethoxysilane 98% (APTES), citric acid monohydrate, sodium dibasic phosphate, phosphate buffer saline tablets (PBS, pH 7.4), 1-ethyl-3-(3-dimethylaminopropyl)-*N*-carbodiimide hydrochloride (EDC) and *N*-hydroxysuccinimide (NHS) were purchased from Sigma-Aldrich, UK. Ammonium hydroxide solution (NH_4_OH, 28–30%) was obtained from Acros Organics, USA. Ethanol absolute (99.5%) and 2-(*n*-morpholino)ethanesulfonic acid (MES, 0.2 M, pH 5.7) were obtained from Fisher Scientific, UK. Amine-modified black hole quencher 2® (BHQ2) was purchased from Biosearch Technologies, USA. Carboxyethylsilanetriol sodium salt, 25% g g^−1^ was purchased from abcrGmbH, Germany.

### Synthesis of size-tuneable silica nanoparticles

Into a scintillation vial, 16.75 mL of absolute ethanol was mixed NH_4_OH (28–30% w/v) with a volume specified in Table 1 (ESI†) to prepare SNPs with diameters ranging from 20 to 200 nm. Following this, 500 μL of TEOS was added dropwise to this mixture (ethanol–NH_4_OH), with continuous stirring for 24 hours at room temperature. To collect SNPs, dialysis and freeze drying was used for nanoparticles <50 nm while centrifugation was used to collect SNPs > 50 nm at 6000 rpm for 30 minutes, followed by washing for three times with 30 mL of absolute ethanol. After the final wash, the suspensions containing SNPs were transferred into a 50 mL rounded bottom flask to evaporate ethanol using a rotary evaporator at 30 °C and the dried nanoparticles were collected and stored at 4 °C. To prepare SNPs with nanoparticle diameter larger than 200 nm, the same procedure described above was used followed by further dropwise addition of 1.5 mL of TEOS after the first 24 hours to each vial containing the specified amounts of NH_4_OH, with continuous stirring for further 24 hours at room temperature. Following this, nanoparticle collection, purification and drying were performed as described using centrifugation.

### Synthesis of fluorescent SNPs, pH nanosensors, core–shell models

Prior to nanoparticle synthesis, fluorophore conjugation to APTES was performed^43^ using an accurately weighed 1 mg of the succinimidyl ester derivative of each fluorophore (OG, FAM or TAMRA), dissolved separately into 1 mL of absolute ethanol, stirred overnight with 20 μL of APTES (98%), in the dark and stored in the dark at 4 °C until further. The protocol for the synthesis of SNPs was followed using 0.75 mL of NH_4_OH, before the addition of TEOS (500 μL), a volume of fluorophore–APTES conjugates (100 μL), was added dropwise to the ethanol–NH_4_OH mixture with continuous stirring for 24 hours in the dark. Following this, 500 μL of TEOS was added dropwise to the reaction mixture with stirring for further 24 hours and the same procedure for SNPs synthesis was followed.

#### To fabricate the pH-sensitive nanosensors

40 μL of OG–APTES, 40 μL of FAM–APTES and 60 μL of TAMRA–APTES were used and the procedure for the synthesis of fluorescent SNPs, described above, was followed.

#### Synthesis of model 1, 2 and 3

Model 1 was synthesised using APTES–TAMRA conjugate (20 μL), added dropwise to the ethanol–NH_4_OH mixture, prepared using 5.8 mmol L^−1^, 0.75 mL. The same procedure for the synthesis of fluorescent SNPs was followed. For the preparation of model 2 and 3, the same nanoparticle batch of model 1 was used using an accurately 10 mg of model 1, transferred into separate scintillation vials and suspended into 1 mL of absolute ethanol. Two plain silica shells, with different thickness, were added to model 1 using either 30 μL or 50 μL of NH_4_OH (28–30% w/v), added to the scintillation vials containing the 10 mg of model 1, with continuous oscillation, followed by consecutive three additions of 17 μL of TEOS with 15 minutes time intervals between each addition, as reported.^47^ After 24 hours, the contents of the vials were centrifuged and washed three times with 1 mL of PBS, pH 7.4 and nanoparticles were collected and used for surface modification, activation and binding to BHQ2, as described below.

### Carboxylic acid surface modification, EDC/NHS activation and binding to amine-BHQ2 ^47^

To modify the nanoparticle surface of model 1, accurately weighed 10 mg of the 75 nm TAMRA-SNPs was suspended into 1 mL of PBS buffer. Model 2 and 3, were directly used after their preparation as in the previous step. Following this, 40 μL of carboxyethylsilanetriol sodium salt (25% g g^−1^) was added to each vial containing the corresponding nanoparticle model and the mixtures were gently stirred for 24 hours in the dark. The resultant carboxylic acid modified SNPs were centrifuged and washed with 1 mL of PBS for three times while the last wash was performed using 1 mL of 0.2 M MES buffer, pH of 5.7. Following this, 30 μL of freshly prepared EDC and NHS solutions (500 mg mL^−1^ prepared separately in MES buffer, pH 5.7) were added to each vial (model). The reaction mixtures were stirred for 30 minutes followed by centrifugation and washing, three times, with 1 mL PBS buffer.

For BHQ2 binding, 120 μL of BHQ2 (1 mg mL^−1^, prepared in ethanol) was added to the EDC/NS activated SNPs models, with continuous stirring for 24 hours, followed by centrifugation and several washings with 1 mL of PBS. The nanoparticle suspension of each model, linked to BHQ2, were collected in PBS (1 mL) and used for measurement of fluorescence intensity/quenching of TAMRA, against blank samples of each batch treated similarly without binding to BHQ2.

### Nanoparticle/nanosensor characterisation

The hydrodynamic diameter and zeta potential measurements were performed using Malvern Zetasizer Nano II DLS instrument. For the hydrodynamic diameter measurements, A 20 μL of the original nanoparticle suspensions was diluted with 3 mL of deionised water. Zeta potential measurements were performed using a zeta potential cuvette of 0.1 mg mL^−1^ of SNPs suspended into 1 mL of 10% v/v PBS. Transmission electron microscopy (TEM) (FEI Tecnai 12, Biotwin) imaging was performed using 0.1 mg mL^−1^ of nanoparticle suspension, prepared in water, at a voltage of 100 kv. All the TEM images were analysed using ImageJ software to determine the median diameter and size distribution. All fluorescence measurements were performed using a Varian Cary Eclipse fluorescence spectrophotometer at the selected excitation and emission maxima of each fluorophore. For nanosensors calibration, 0.5 mg mL^−1^ of nanosensors were suspended in citric acid monohydrate/sodium dibasic phosphate buffer, prepared with different pH values from pH 2.5–8.5 and fluorescence measurements were performed. Fluorescence intensities of OG and FAM were detected at 520 nm after excitation at 488 nm while the fluorescence intensity or quenching of TAMRA was recorded at 577 nm after excitation at 545 nm. The ratio between the fluorescence intensity of OG/FAM and TAMRA, detected in the different pH values, was calculated and used to construct the ratiometric fluorescence calibration curve, against blank buffer samples.

To investigate the fluorescence quenching using SNPs models, the relationship between varying amounts of BHQ2 and the fluorescence intensity of model 1 was recorded after binding of the EDC/NHS activated SNPs to different amounts of BHQ2 (0.02 to 0.52 μmol L^−1^, 10 to 250 μL of 1 mg mL^−1^), followed by centrifugation and washing as described previously. The fluorescence quenching of TAMRA-SNPs after binding to BHQ2 was measured against blank samples treated similarly without the reaction with BHQ2. All fluorescence quenching measurements were detected for the corresponding SNPs/core–shell model against blank sample treated similarly and the percentage of fluorescence quenching was calculated.

## Author contributions

ME developed the protocol for the preparation of size tuneable nanoparticles, synthesised the pH-sensitive nanosensors and conducted DLS and fluorescence measurements. AS produced size-tuneable silica nanoparticles using the protocol developed and conducted TEM analysis (size tuneable and core shell particles). ME, AS and VMC prepared figures and tables. ME produced core–shell particles and optimised quenching experiments. ME, VMC, SJBT and JWA prepared manuscript. ME and VMC produced ESI.† ME, VMC, SJBT and JWA edited manuscript for publication. All authors contributed to scientific planning and direction.

## Conflicts of interest

The authors declare no conflict of interest.

## Supplementary Material

RA-008-C8RA05929B-s001
