# Supplementary material for: Enhanced distance-dependent fluorescence quenching using size tuneable core shell silica nanoparticles
Source: RSC Adv. 2018 Oct 19;8(62):35840–8. doi: 10.1039/c8ra05929b (PMC9088216; doi:10.1039/c8ra05929b)
Supplement: RA-008-C8RA05929B-s001 [file RA-008-C8RA05929B-s001.pdf]

Electronic Supplementary Material (ESI) for RSC Advances.
This journal is © The Royal Society of Chemistry 2018

SUPPORTING INFORMATION

Enhanced Distance-Dependent Fluorescence Quenching Using Size Tuneable Core Shell Silica Nanoparticles

Mohamed M Elsutohy [1,2], Amjad A Selo [1], Veeren M Chauhan [1], Saul JB Tendler [1,3] and Jonathan W Aylott [1]*

[1] Boots Science Building, School of Pharmacy, University Park Campus, University of Nottingham, Nottingham, NG7 2RD, UK. [2]Department of Pharmaceutical Analytical Chemistry, Faculty of Pharmacy, Al-Azhar University, Assiut 71524, Egypt, [3]Vice-Chancellor's Department, University of York, Heslington, York, YO10 5DD, UK.

*Address correspondence to: jon.aylott@nottingham.ac.uk

1. Fabrication of pH-sensitive nanosensors

Ratiometric, fluorescent pH-sensitive nanosensors, consisted of 75 nm SNPs, were fabricated using the protocol described for the synthesis of fluorescent silica nanoparticles (SNPs) (Figure S1 A). Fluorophores were separately conjugated to an excess molar amounts of APTES to ensure that all fluorophore molecules were conjugated to prepare fluorophore-conjugate that was used to synthesise nanosensors. The effective dynamic pH range of nanosensors can be extended by selecting the type of the pH-sensitive fluorescent dyes, linked to the nanoparticle matrix[1]. Two pH-sensitive fluorescent dyes, Oregon green (OG) and 5-(6)-carboxyfluorescein (FAM) and a pH-insensitive reference fluorophore (TAMRA) were selected. Selection of such two pH-sensitive fluorescent dyes (OG and FAM) was based on the fact that they exhibit the same excitation and emission wavelengths (490 nm and 520 nm, respectively) while they have different pK_a values (pH 4.6 and 6.4, respectively); this extends the effective dynamic range of the measurements. In addition, TAMRA was selected as a pH-insensitive fluorophore that exhibits excitation and emission at 545 nm and 575 nm, respectively, to permit ratiometric fluorescence pH measurements[2].

To optimise the maximum nanosensor response, the effect of different ratios of OG: FAM, linked to APTES, was investigated. Nanosensor calibration was performed in citric acid monohydrate/sodium dibasic phosphate buffer solutions (pH from 2.5 to 8.5) and the ratio between fluorescence intensity of OG/FAM, measured at 520 nm, and TAMRA, recorded at 575 nm, was calculated versus the corresponding pH value. As the ratio of OG: FAM varied, the effective dynamic range of nanosensors was changed (Figure S1 B). For nanosensors that incorporated OG only (100% OG: 0% FAM), the effective dynamic pH range of nanosensors (pKa±1) was controlled by the activity of OG (pKa ~4.6) with an effective response from pH 3.5 to 5.5 where pH = pKa±1. Similarly, the responsive pH range of nanosensors encapsulating FAM only was from pH 5.5 to 7.5, as the activity of these nanosensors was controlled by the pKa of FAM (~6.4). Tuning the ratio of OG: FAM produced nanosensors with tuneable effective pH responses while the maximum extended dynamic response was observed for nanosensors incorporating OG: FAM in a ratio of 1:1 (50%: 50%). Such nanosensors were able to detect pH changes between pH 3.5 and 7.5 (linear area of the sigmoidal calibration curve), Figure S1 C and D.

Dye leaching is a common problem, associated with optical nanosensors that are physically entrap fluorophores within nanoparticle matrix [3, 4]. Nanosensors developed in this study showed no or negligible dye leaching from the silica matrix as confirmed by detecting the fluorescence signals of nanosensors in suspension and comparing such values to that recorded in the supernatant post nanoparticle centrifugation. Fluorescence recorded in the supernatant did not exceed 2% of the original fluorescence signal, recorded for nanosensors in suspension. Furthermore, the fluorescence signal intensity of each fluorophore was recovered after re-suspending nanosensors pellets again. Additionally, fluorescence stability study was performed by recording the fluorescence signals for each fluorophore over periods ranging from 5 minutes

to 48 hours. As illustrated in Figure S1-E, there is a slight decrease in the recorded fluorescence intensity of fluorophores. However, the ratio of the fluorescence, recorded over time, remained without any significant change and was within 0.1 unit, Figure S1 F. This illustrates the advantageous use of ratiometric fluorescence measurements compared to direct fluorescence detection, which may give inaccurate data due to fluorescence signal fluctuations.

While previously reported silica-based, fluorescent, pH-sensitive sensors that were synthesised using a single pH-sensitive fluorophore [5] exhibited a limited effective measurement range that was restricted to one pH unit either side of their calculated pK_a [1, 5]; similar to nanosensors linked to either OG or FAM prepared in this study (Figure S1-B). Nanosensors optimised in this study exhibited an extended effective range up to four pH unites by optimising the ratio of the pH-sensitive fluorophores (pH 3.5 to 7.5). Further, covalent conjugation of fluorescent dyes to nanoparticle matrix could overcome the drawback of attaching dyes to nanoparticle surface; *e.g.* photobleaching and toxicity, which has been previously reported for a silica-based pH-sensitive nanosensors [6].

2. Core-shell synthesis, carboxylic acid functionalisation [7], EDC/NHS activation and binding to amine-BHQ2

Silica shells with different thickness was prepared using a previously reported method [7]. The control of the silica shell thickness was demonstrated using a constant amount of TEOS with varying amounts of NH_4OH to add further layers of silica around nanoparticles [7, 8].

Nanoparticle surface modification was performed using carboxyethylsilanetriol sodium salt (25% w/w) that able to add a carboxylic acid group to the nanoparticle surface[7]. This modification was peroforemd to facilitate binding to amine-modiefed black hole quencher 2 (BHQ2) molecules. Measurements of zeta potential were used to evaluate this surface functionalisation. The results revealed that the zeta potential value of non-functionalsied SNPs was -31±5 mV which was significantly increased in the negative direction to -55±3 mV post carboxylic acid nanoaprticle surface functionalisedation, Figure S2-A. In addition, this modification step did not markedly influence the size of SNPs as the measured hydrodynamic diameters of carboxy-SNPs were 79 ± 9 nm which is almost in the same size range of the non-functionalised SNPs (75 ± 12). To optimise the quantitiy of carboxyethylsilanetriol sodium salt required for maximum surface modification, different volumes of carboxyethylsilanetriol sodium salt (25% w/w), ranging from 20 µL to 200 µL (0.0336 mmol to 0.336 mmol) were used. Zeta potential values of the resulting SNPs functionalised with carboxylic acid group were measured after functionalisation and compared to non-functionalised nanoaprticles. The results showed that varying volumes of carboxyethylsilanetriol sodium salt did not markedly influence the zeta potential value which remained at about -50 ± 11 mV (Figure S2-B). The obtained results were in agreement to that found in the literature that stating that varying quantitiites of CS did not influence the functionalisation process and suggest that the surface of SNPs could be fully saturated with carboxylic acid moiety [7, 9]. As a result, for surface functionalisation of SNPs with carboxylic acid groups, 40 µL (0.067 mmol) was used for functionaliseation as reported[7].

Carboxylic acid group, grafted onto nanoparticle surface, was further activated using 1-Ethyl-3-(3-dimethylaminopropyl)-N'-carbodiimide hydrochloride (EDC) and N-hydroxysuccinimide (NHS) to increase binding efficiency to a primary amine modified molecule [10-12], e.g. amine-BHQ2. This activation technique is frequently used in particle and surface conjugation procedures, making this process the most popular activation technique in use today [11]. The EDC/NHS coupling chemistry is based on using the zero-length carbodiimide crosslinker (EDC) to activate the carboxylic acid group and form an amine reactive o-acylisourea intermediate that spontaneously reacts with the primary amine group to form an amide bond. While this reactive intermediate is highly unstable and rapidly hydrolysed in aqueous solutions, NHS or sulfo-NHS is coupled with EDC to react with the resultant unstable intermediate, , increasing the stability, water solubility and improve the efficiency of the conjugation[11, 13, 14], Figure S3. EDC/NHS activation reaction is highly sensitive to the pH of the medium while the most efficient reaction occurs within pH from 4.7 to 6.0 in primary amine-free and carboxylate-free buffers, such as 2-(n-morpholino) ethanesulfonic acid (MES) buffer, with a pH from pH 5 to 6, is usually used as the medium for the activation reaction [11].

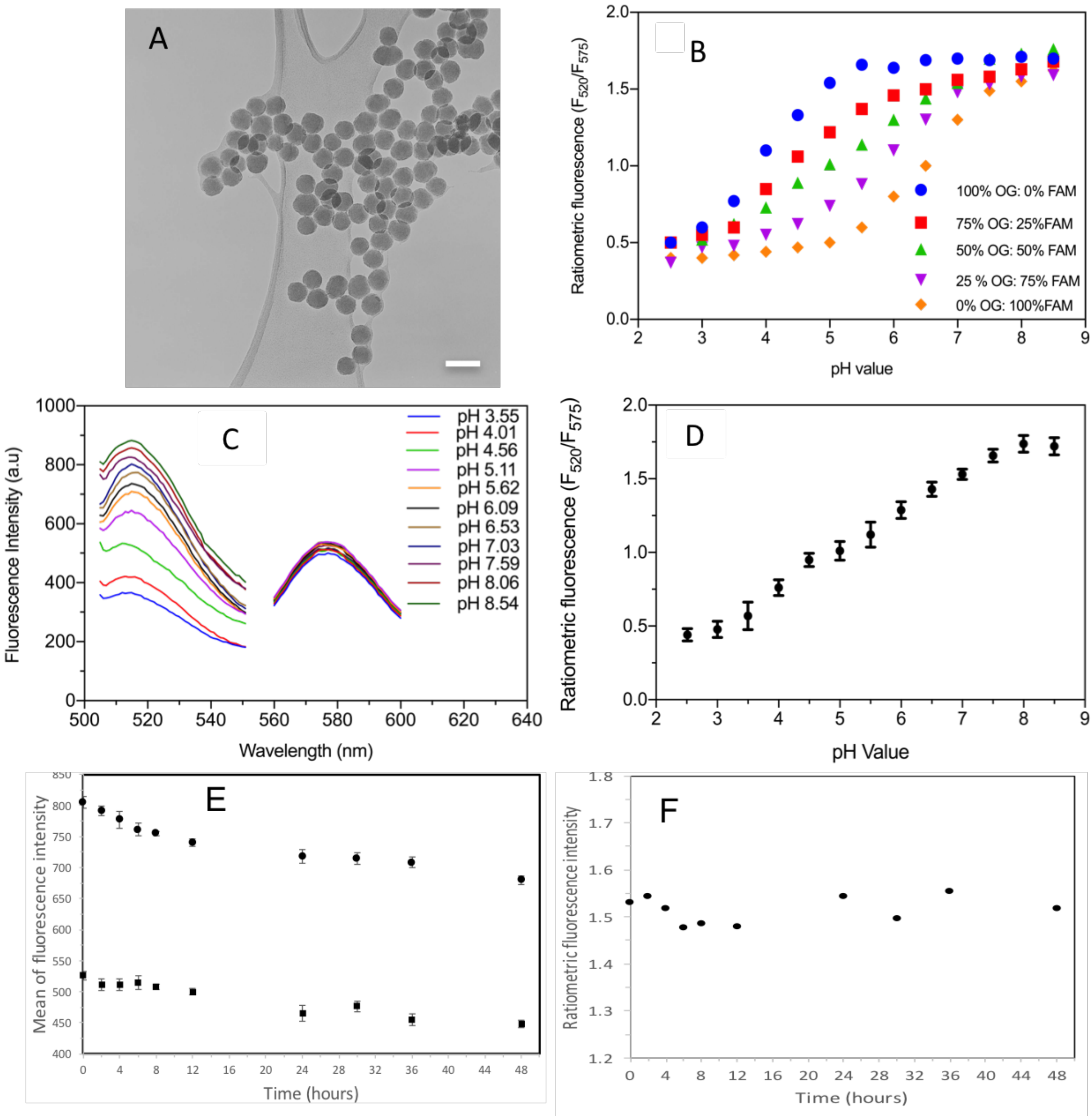

Figure S1: (A) TEM image for the silica-based pH-sensitive nanosensors, optimised in this study, scale bar = 100 nm. (B) Ratiometric fluorescence calibration curves of pH-sensitive nanosensors, fabricated from SNPs covalenly linked to different ratios of two pH-sensitve fluorphores (OG:FAM) and a constant amount of a reference pH-insensitive fluorophore (TAMRA). (C) Fluorescence spectra of nanosensors containing the optimised ratio of OG: FAM (1:1 = 50%50%) and TAMRA. Fluorescence of OG and FAM detected at 520 nm while for TAMRA at 575 nm. (D) Calibration curve of the optimised nanosensor suspended in a wide range buffer from pH 2.5 to pH 8.5 with an efffective range from pH 3.5 to 7.5 (linear area of the sigmoidal calibration curve). (E) Stability of fluorescence signal of nanosensors recorded at 520 nm for OG, FAM and at 575 nm for TAMRA, nanosensors suspended in PBS buffer. (F) Ratiometric fluorescence measurment of the signals recorded in (E).

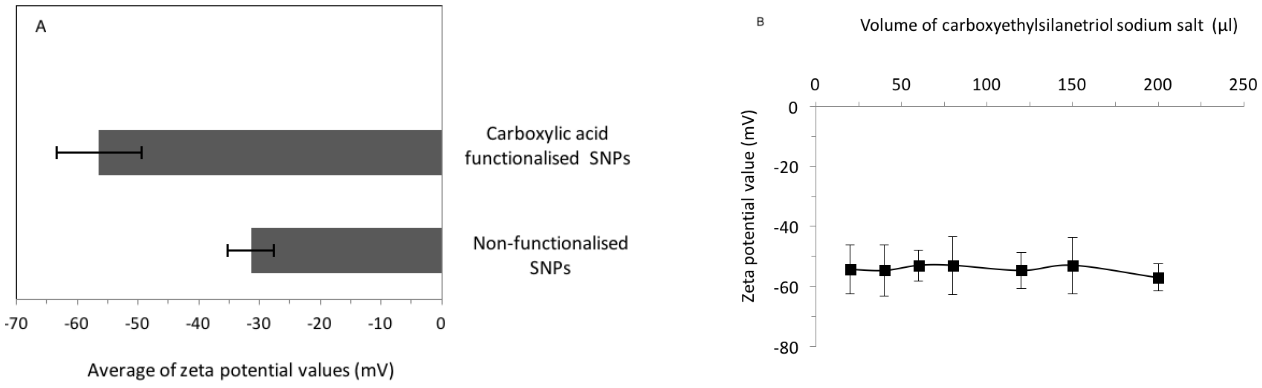

Figure S2: (A) Zeta potential values of non-functioanlsied SNPs and post carboxylic acid surface functionalisation. (B) The influence of varying volumes of carboxyethylsilanetriol sodium salt (25% w/w) on zeta potential.

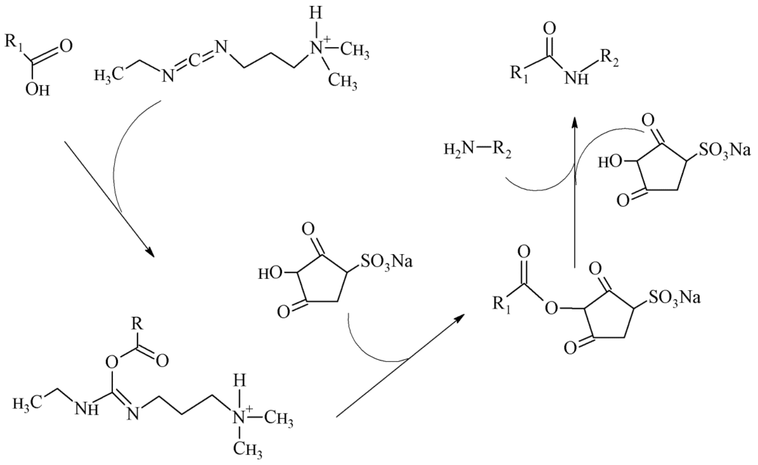

Figure S3: A schematic for the chemical reaction between carboxylic acid containing molecule (e.g: carboxylic acid functionalised SNPs) with amine-molecule (e.g: amine-BHQ2) using the EDC/sulfo-NHS coupling chemistry to increase the reaction efficiency.

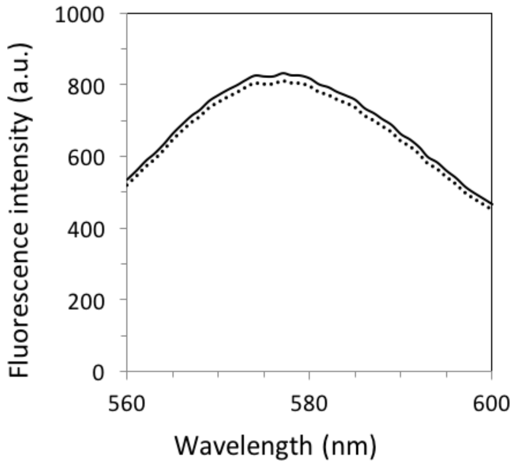

Figure S4: Fluorescence spectra of model 1 (TAMRA-SNPs), prepared without carboxylic acid surface functionalisation or EDC/NHS activation (solid line), and for the same model mixed with amine-BHQ2 for 24 hours (dashed line).

Table 1: The volume of NH_4OH (28-30% w/v) that were attempted to synthesise size-tuneable SNPs (20 to 200 nm) and the corresponding nanoparticle yield

Volume of NH_4OH (mL)	Average hydrodynamic diameters (nm)	Approximate nanoparticle yield
**0.3 mL (2.3 mmol/L)**	20 ± 7	Below 15 mg
**0.5 mL (3.9 mmol/L)**	45 ± 12	~ 35 mg
**0.75 mL (5.8 mmol/L)**	75 ± 9	50 – 100 mg
**0.85 mL (6.5 mmol/L)**	93 ± 10	~100 mg
**1.0 mL (7.3 mmol/L)**	120 ± 11	~ 150 mg
**1.25 mL (9.6 mmol/L)**	140 ± 19	~ 200 mg
**1.5 mL (11 mmol/L)**	152 ± 5	~ 300 mg
**1.75 mL (13 mmol/L)**	180 ± 17	~400 mg
**2.0 mL (14.6 mmol/L)**	192 ± 8	>500 mg
**3.0 mL (23.1 mmol/L)**	200 ± 22	>500 mg
**4.0 mL (29.2 mmol/L)**	204 ± 13	>500 mg
**5.0 mL (36.5 mmol/L)**	208 ± 16	>500 mg

References

1. V. M. Chauhan, G. Orsi, A. Brown, D. I. Pritchard and J. W. Aylott, *ACS Nano*, 2013, **7**, 5577-5587.
2. M. M. Elsutohy, V. M. Chauhan, R. Markus, M. A. Kyyaly, S. J. B. Tendler and J. W. Aylott, *Nanoscale*, 2017, **9**, 5904-5911.
3. R. Gupta and N. K. Chaudhury, *Biosens Bioelectron*, 2007, **22**, 2387-2399.
4. S. M. Borisov and O. S. Wolfbeis, *Chem Rev*, 2008, **108**, 423-461.
5. T. Doussineau, S. Trupp and G. J. Mohr, *J Colloid Interface Sci*, 2009, **339**, 266-270.
6. S. C. Hu, L. Sun, M. X. Liu, H. D. Zhu, H. L. Guo, H. M. Sun and H. H. Sun, *New Journal of Chemistry*, 2015, **39**, 4568-4574.
7. J. Labéguerie-Egéa, H. M. McEvoy and C. McDonagh, *Journal of Nanoparticle Research*, 2011, **13**, 6455-6465.
8. Andrew Burns, Prabuddha Sengupta, Tara Zedayko, Barbara Baird and a. U. Wiesner, *Small*, 2006, **2**, 723-726.
9. R. P. Bagwe, L. R. Hilliard and W. Tan, *Langmuir*, 2006, **22**, 4357-4362.
10. Marye Anne Fox and a. J. K. Whitesel, *Organic Chemistry*, Jones and Bartlett International Publishers, Oxford, UK, Third edn., 2004.
11. G. T. Hermanson, *Bioconjugate Techniques*, Elsevier, Oxford, UK, Second edn., 2008.
12. S. S. Wong, *Chemistry of Protein Conjugation and Cross-Linking*, CRC Press, Florida, USA, 2000.
13. J. Genzer, *Soft Matter Gradient Surfaces: Methods and Applications*, Wiley & Sons Ltd, New Jersy, USA, 2011.
14. K. S. Rao, S. U. Rani, D. K. Charyulu, K. N. Kumar, B. K. Lee, H. Y. Lee and T. Kawai, *Anal Chim Acta*, 2006, **576**, 177-183.
